# Connecting the experiences of persons with disabilities and social workers in Nigerian care institutions regarding COVID-19 vaccine uptake: a qualitative descriptive-interpretive design

**DOI:** 10.3389/fpubh.2024.1466313

**Published:** 2024-10-09

**Authors:** Farah Naz Rahman, Anthony Obinna Iwuagwu, Christopher Ndubuisi Ngwu, Michael Ebe Kalu, Amani Kasherwa, Mohammad Rocky Khan Chowdhury, Manzur Kader

**Affiliations:** ^1^International Centre for Diarrhoeal Disease Research Bangladesh (icddr, b), Dhaka, Bangladesh; ^2^School of Public Health and Preventive Medicine, Monash University, Melbourne, VIC, Australia; ^3^Department of Social Work, University of Nigeria, Nsukka, Nigeria; ^4^School of Kinesiology and Health Science, York University, Toronto, ON, Canada; ^5^School of Nursing, Midwifery, and Social Work, The University of Queensland, St Lucia, QLD, Australia; ^6^Department of Medical Science, School of Health and Welfare, Dalarna University, Falun, Sweden

**Keywords:** disability, rehabilitation homes, social work, COVID-19, vaccine knowledge, Nigeria

## Abstract

**Background:**

During the COVID-19 pandemic, persons with disabilities (PWDs) have faced additional disadvantages that have exacerbated their physical and mental health challenges. In Nigeria, where cultural, religious, and informational barriers persist, understanding these factors is critical for improving health interventions, including vaccine uptake among PWDs.

**Methods:**

This study employed a qualitative descriptive-interpretive design to explore the perceptions of PWDs regarding the COVID-19 pandemic and the vaccine, alongside social workers’ views on their roles in facilitating vaccine uptake. We conducted in-depth semi-structured telephone interviews with 20 participants, comprising 16 PWDs and four social workers in Nigerian rehabilitation homes. Data were analyzed using critical thematic analysis to identify key themes influencing attitudes toward the pandemic and vaccine uptake.

**Results:**

The study uncovered significant barriers to COVID-19 vaccine uptake among PWDs, primarily driven by mistrust in government initiatives, widespread conspiracy theories, and deeply held cultural and religious beliefs. Additionally, while social workers played crucial roles as community surveillance officers, in-house educators, and community referral agents, their interventions lacked specific strategies aimed at increasing vaccine uptake among PWDs. Their efforts were more focused on addressing the psychological impacts of the pandemic rather than fostering behavioral changes toward vaccine acceptance.

**Conclusion:**

To enhance COVID-19 vaccine uptake among PWDs in Nigerian rehabilitation homes, targeted interventions that address the identified barriers are essential. These should include trust-building measures, culturally and religiously sensitive communication strategies, and tailored educational programs by social workers. Moreover, training social workers in specific, evidence-based strategies to increase vaccine uptake is crucial for mitigating the pandemic’s impact on this vulnerable population.

## 1 Introduction

Globally, over 1 billion people live with disabilities [PWD] (1). Persons with disabilities (PWD) include individuals with long-term physical, mental, intellectual, or sensory impairments that interact with various barriers hindering their full and effective participation in society on an equal basis with others (2). PWD is far less inclined to access quality health care and is more likely to experience severe health needs, discriminatory practices, and prejudice (3). The 2019 Coronavirus [COVID-19] pandemic is expected to compound the already existing inability to access quality healthcare because many of the health and social services provided for people with disabilities have been interrupted (1). PWDs are further disadvantaged, leading to poor physical and mental health (4, 5). In Nigeria, PWDs already have limited access to health and social care services, and the COVID-19 prevention strategies, e.g., the lockdown, have further made access to these services difficult.

In Nigeria, over 29 million persons live with various types of disabilities (5). The five most prevalent disabilities in Nigeria (in descending order) are visual impairments, auditory impairments, physical impairments, intellectual impairments, and communication impairments (6). About 7% of household members over the age of 5 (and 9 percent of those 60 and older) have some challenges in a minimum of one functional domain, such as seeing, hearing, communication, cognition, walking, or self-care; and 1 percent have severe difficulty or are unable to work in at least one domain (7). In Nigeria, PWDs are partially ignored and are treated as a welfare or charity case (8). Therefore, programs to empower and socially include PWDs are limited in Nigeria, resulting in PWDs seeking ways to empower themselves, including panhandling (asking people on the street for food or money) on Nigeria’s streets. Traditionally in Nigeria, PWDs live at home with their relative (9). Some are admitted into a rehabilitation home, either temporarily or permanently. Those admitted permanently are often those whose relatives have abandoned or do not have relatives who can support them, whereas those admitted temporarily usually require professional social and physical rehabilitation services. Nevertheless, PWDs in Nigeria account for the highest percentage of individuals living below the poverty line, placing them at the lowest socioeconomic strata (5, 10). These accumulated disadvantages put them at the risk of contracting coronavirus.

COVID-19 is a novel virus that was first identified in Wuhan, China. The transmission of COVID-19 from one person to another was rapid, as World Health Organization declared the virus a pandemic just after 3 months of its first transmission. The spread of the COVID-19 virus continues but slower, maybe because people have started observing the safety measures and vaccine administration. While other countries’ vaccine administration began in early Jan. 2021, Nigeria’s vaccine administration started in late March 2021. According to the African Union Center for Disease Control and prevention, the priority group is healthcare workers (11); this puts older adults and PWDs in an unfortunate situation as they are at high risk of contracting, developing complications, and dying from COVID-19. PWDs are more likely to get infected because of the underlying medical conditions, congregated living settings, systemic health, and social inequities (12). Besides, PWDs, especially with intellectual disabilities, may have trouble understanding information or practicing prevention measures and difficulty communicating symptoms of COVID-19 (13). COVID-19 strategies to prevent transmission may be reinforced again in the Nigerian context as vulnerable groups, including older adults and PWDs, have refused to take the vaccine (14–16).

Globally, several studies have explored the PWD’s perception of COVID-19 on their wellbeing, access to information concerning the COVID-19 during the lockdown. Most of the studies are either quantitative or reviews and focused on the developed countries, with few opinion papers in developing countries, e.g., Nigeria. For instance, Lebrasseur et al. (17) conducted a rapid review that explored the impact of the COVID-19 pandemic on PWDs. They included 11 articles that reported decreased access to health/social service and social and lifestyle changes, including mood changes and decreased physical activities among PWDs. Similarly, in an online survey report, PWDs residing in the Netherlands reported unstable emotions such as fear and anxiety during COVID-19 lockdown (18). In Spain, Amor and colleagues’ (19) surveyed 582 people living with intellectual disability and reported that lockdown harmed participants’ emotional wellbeing, occupations, and access to information concerning COVID-19 was good. In Nigeria, evidence on the impact of COVID-19 on PWDs is limited and mostly on opinion papers (20, 21) with no empirical data. We found a cross-sectional study investigating demographic factors, attitudes, and knowledge of persons with special needs during COVID-19 in Nigeria (22). They reported that persons with special needs have high knowledge about the symptoms, prevention, and control of COVID-19, and 52.8 and 55.6% of the participants reported that it is hard to get palliatives or financial support from others and feeling frustrated by the uncaring attitude of the government toward them during the COVID-19 lockdown. While this study’s findings highlighted the level of knowledge and attitude of COVID-19 characteristics and lockdown perception among persons with special needs, their study population included a wide variety of PWDs regardless of residential settings. PWDs in congregated settings present unique health and social inequities that may differentiate them from those living at home with relatives. Therefore, a qualitative method is needed to explore the perceptions of the COVID-19 pandemic and vaccines among PWDs residing in rehabilitation homes.

Social workers, through their competency skills, are recognized as front-line workers in the fight of COVID-19. However, their roles in providing and advocating for PWDs are rarely highlighted in Nigeria. Besides, social workers are trained to be resilient and provide care or relief services during disasters, such as pandemics (23). Interestingly, the International Federation of Social Workers [IFSW] recommended specific roles social workers should play during the COVID-19 pandemic, including ensuring that the most vulnerable (e.g., PWDs) are actively involved in planning and response across various communities, providing alternatives to care for vulnerable individuals while facilitating observance of the COVID-19 prevention measures and vaccine uptake (24). While Ajibo et al.’s (25) study has explored the role of social workers in cushioning the effect of COVID-19 in Nigeria, no study explored social workers’ role in increasing vaccine uptake among PWDs in rehabilitation homes. Our study aimed to understand social workers’ role in increasing vaccine uptake among PWDs residing in rehabilitation homes.

Given COVID-19 lethality and the different susceptibility of PWDs, it is crucial to explore their experiences and recognize the concerns that are deemed more critical during the lockdown in a low resource country like Nigeria. Besides, since communication and supports are pivotal in preserving public health and equally crucial to preventing the spread of the disease, understanding social workers’ responses to increasing vaccine uptake among PWDs in rehabilitation homes is also helpful. Therefore, the primary aim of this study was to explore how PWDs’ perceptions of the COVID-19 pandemic informed vaccine uptake in Nigeria. The secondary aim was to explore the social workers’ perception of increasing vaccine uptake among PWDs in rehabilitation homes.

## 2 Materials and methods

We employed a qualitative descriptive-interpretive design to guide the data collection and analysis (26). This design allowed us to interpret participants’ perceptions on COVID-19 and COVID-19 vaccines uptake by identifying the prominence of ideologies, power relations, and status-based hierarchies. Descriptive-interpretive design emphasizes detailed descriptions and interpretation of participants’ experiences, allowing for nuanced understanding of complex phenomena like vaccine acceptance within a specific cultural context. Given the multifaceted nature of disability and its intersection with healthcare access and social support, this approach facilitates capturing the diverse perspectives and lived experiences of both persons with disabilities and social workers. Unlike other qualitative methods like phenomenology or grounded theory, which may focus on abstract concepts or theoretical frameworks, the descriptive-interpretive design prioritizes rich, contextualized data collection and analysis, making it well-suited for exploring the unique challenges and facilitators influencing vaccine attitudes and uptake in this population in Nigeria. Additionally, its flexibility in data collection methods, such as interviews and observations, aligns well with the need for inclusive and participatory research approaches when working with persons with disabilities and their support networks.

Data collection involved semi-structured interviews with PWD and social workers in three rehabilitation homes. Ethical approval was obtained from the Health Research Ethics Committee, University of Nigeria Teaching Hospital Ituku-Ozalla (NHREC-1RB00002323). This approval ensures that the research adheres to established ethical guidelines, safeguarding the rights and well-being of all participants involved. Prior to their involvement, participants were fully informed about the study’s objectives, procedures, and potential risks, and they provided voluntary informed written consent. Additionally, anonymity and confidentiality of the participants were maintained throughout the data collection, analysis, and scientific writing procedure. We followed the Consolidated Criteria for Reporting Qualitative Research (COREQ) (27) in reporting this study.

### 2.1 Study setting/recruitment

We conducted this study in three rehabilitation homes located in the Anambra, Enugu, and Taraba States. These three rehabilitation homes are private care institutions in Nigeria where persons living with disabilities are accommodated, provided for, and receive physical and social rehabilitation, including physical and occupational therapy, vocational training, counseling, re-integration training programs. The homes accommodate residents with physical disabilities only, and among them residents with visual impairments have targeted support and services for their different needs than others. At the time of this study, there were 104 residents and eight social workers in the three homes. Participants were recruited through snowballing, where participants who participated in the study informed someone eligible to participate. Initial contacts were established through trusted relationships developed with some residents during previous voluntary work by some of the research team members at these homes. Participants were encouraged to refer others by explaining the importance of the study and how additional insights would enhance the understanding of vaccine acceptance among their community. To ensure a diverse and representative sample, efforts were made to include participants with various types of disabilities and different backgrounds (age, gender, education, and religion), ensuring perspectives from both residents and social workers across the three states were represented in the study.

### 2.2 Sampling

We employed criterion-based purposive sampling in selecting our participants. PWD participants were included if they are (a) 18 years or older, (b) able to consent and communicate in English, Nigerian Pidgin English, Hausa, and Igbo language (which are dominant languages in the states the PWD were recruited from), (c) self-identified as having a disability, and (d) resides in the center. Social worker’s participants were included if they have (a) at least a post-graduate degree in social work and practicing and (b) 3 years’ experience in working with PWD residing in these homes.

The United Nations Convention on the Rights of Persons with Disabilities (CRPD) provides a broad, inclusive definition of disability, focusing on a human rights perspective. It defines persons with disabilities as those with long-term physical, mental, intellectual, or sensory impairments that, when combined with societal barriers, may limit their full participation in society (28). These impairments include physical (affecting mobility, coordination, or bodily functions), mental (impacting cognitive or psychological health), intellectual (affecting cognitive development), and sensory (affecting hearing or vision) (28). For our study, we included participants with physical impairments or vision-related sensory impairments, considering the feasibility of conducting in-depth telephone interviews.

By selecting individuals who met specific criteria, the study ensured that the participants were most suitable for understanding the study concepts, relating to the questions, and effectively expressing their opinions. Additionally, purposive sampling enabled us to employ a targeted approach to identifying and gathering relevant data from those whose insights were most meaningful for the study. Because data collection and analysis were done concurrently, we stopped interviewing after reaching data saturation and sufficiency, explaining why a smaller proportion of PWDs was interviewed (29). The final number of included participants show a balanced representation of residents with physical disabilities (PD) and visual impairments (VI), ensuring that the study captures varied challenges and attitudes toward the COVID-19 vaccine. The inclusion of both male and female participants, a range of ages (from 18 to 48), and individuals from different educational backgrounds further enhances the diversity of perspectives. Additionally, the representation of participants from various religious backgrounds (Christianity and Islam) and ethnicities (Igbo and Hausa) across different states (Enugu, Taraba, and Anambra) adds depth to the understanding of cultural and regional influences on vaccine acceptance. This diverse sample allows for a comprehensive exploration of how different disability types and socio-demographic factors affect experiences with COVID-19 and vaccination within the PWD community.

### 2.3 Data collection

We invited each participant to a single, semi-structured telephone interview. Telephone interview was appropriate as it allowed us to obey the social distancing policies during the COVID-19 pandemic. Participants were informed of the study aim, the risks, and benefits of participating in the study, confidentiality, anonymity, and the right to withdraw at any time. All participants provided written and oral consent. We conducted a pilot telephone interview with two PWDs to reflect on the wordings in the questions, reflect how sensitive questions asked would be, and determine appropriate timing for the interviews (data from this pilot were not included in the analysis). The telephone interviews lasting between 40 and 45 min were audio-recorded and conducted in English, or Nigerian Pidgin English by a trained researcher using a semi-structured interview guide developed based on the study aim (see Table 1). The interview guide was self-developed based on the study aim. Only five interviews were conducted in Nigerian Pidgin. We kept reflective and field observation notes throughout the study to enhanced rigor in our study. We identify our “Subjective I’s”—the assumptions and the beliefs the research brought into the research (30) and record in our reflexive notes detailing how these assumptions may influence our data collection analysis. For instance, some of the authors are social workers with a strong passion for professional growth in Nigeria. They wrote their assumption on the role of social workers during the pandemic in Nigeria, consciously returned to this assumption, and reflected how it influences the data collection and analysis.

**Table 1:** Open coding subthemes and closed coding themes.

Coding phase	Open coding	Closed coding
Findings/interpretations	What was repeated, recurrent and forceful in the transcripts? Corrupt government Increasing number of COVID-19 cases to attract international aid No objective implementation of palliatives	What ideologies are recurring, repeated, and forceful? Mistrust on government
	COVID-19 affects only “sinners”—those who do not believe in God. Those who believe in God are immune	Religious and cultural standpoints
	COVID-19 is the sickness of the “elites”—rich COVID-19 is a strategy to make developing countries poorer COVID-19 linked to 5G network	Conspiracy theories perpetuated by the media
	Hand washing is regular in our centers Distancing and lockdown increase our problems Perceived impact of COVID-19 lockdown on persons with disability May not take the vaccine	Poor compliance with COVID-19 because of mistrust of the government Increased hunger and delayed spiritual and physical healing when obeying social distancing and lockdown Increased doubt over COVID-19 vaccine uptake
	Neglected roles of Nigerian social workers to PWD during COVID-19 pandemic	Increased volunteering role play

### 2.4 Data analysis

Data collection and analysis were done simultaneously; this allowed us to use complete variance sampling and ensured data sufficiency (informational redundancy) (31). The translation process from Nigerian Pidgin to English was handled by two researchers (AI, CN) with expertise in both the language and culture, as well as post-graduate degrees in social work and extensive experience in qualitative research. They independently transcribed and translated the interviews into English, then met to compare and reconcile the translations, ensuring accuracy and cultural sensitivity (32). We utilized critical thematic analysis (CTA, Lawless and Chen) (29), an extension of Braun and Clarke (32), in analyzing our data. CTA has two major analytical steps: open coding and closed coding. In open coding, two coders (AI, FNR) independently read the transcript of three PWD and two social workers to have a general sense of the data. Both coders independently re-read the transcript to create early codes and categories based on the three concepts of CTA: recurrence, repetition, and forcefulness (33). For instance, coders independently identify statements that provide similar meaning but are described differently by the participants (recurrence) or words used frequently (repetition). Coders also highlight forcefulness by identifying the tone, volume, and emphasis participants lay on statements. Both coders meet to merge the codes and categories to derive a codebook. Any disagreement was resolved in team meetings. We used the codebook to analyze other transcripts; any new codes and categories that emerged were added to the codebook and documented in the audit trail notes. Themes were developed using closed coding processes. All authors meet three times to interlink the categories with dominant societal questions, such as the misconceptions regarding COVID-19 while paying close attention to the discriminatory and non-inclusive practices nested in power relations in Nigeria (see Table 1). The data was managed in NVivo© Software.

### 2.5 Trustworthiness

We employed several techniques to improve the rigor process in this study. First, we maintain reflexivity by identifying our “Subjective I”—the assumptions we carry into the research during the data collection and analysis (30). Second, we increased this study’s credibility by using double coders at each stage of our analysis. Double coders were selected based on their relevant experience, with one coder being from Nigeria with previous experience working in the home, and the other from outside Nigeria, providing fresh, independent perspectives. We also employed peer-member checking—four independent scholars experienced in CTA analysis examined and provided feedback on the themes, which was incorporated into the final analysis to enhance the study’s credibility (32).

## 3 Results

**Figure 1:**
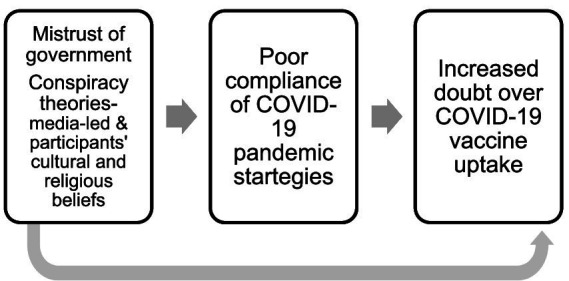
Themes leading to poor compliance of COVID-19 pandemic strategies to perceived poor COVID-10 vaccine uptake.

Twenty participants (16 PWDs and four social workers) participated in this study; see Table 2 for participants’ demographics. Summarily, three themes: mistrust of government, conspiracy theories perpetuated by the media, and participants’ culture and religious beliefs influence poor compliance of COVID-19 prevention strategies, leading to increased doubt over COVID-19 vaccine uptake among PWDs in rehabilitation homes in Nigeria (see Figure 1).

**Table 2:** Demographic information of participants.

S/N	Age (years)	Gender	Highest level of education	Religion	Location (State)	Ethnicity	Disability type
1	21	Female	FSLC	Christianity	Enugu	Igbo	PD
2	27	Male	WASSCE	Islam	Taraba	Hausa	PD
3	25	Male	WASSCE	Christianity	Anambra	Igbo	VI
4	24	Female	WASSCE	Islam	Taraba	Hausa	PD
5	24	Male	WASSCE	Christianity	Anambra	Igbo	PD
6	27	Female	B.Sc	Christianity	Enugu	Igbo	VI
7	20	Female	WASSCE	Islam	Anambra	Igbo	PD
8	18	Female	WASSCE	Christianity	Taraba	Hausa	VI
9	22	Female	FSLC	Christianity	Enugu	Igbo	VI
10	21	Female	FSLC	Christianity	Enugu	Igbo	PD
11	23	Male	FSLC	Christianity	Anambra	Igbo	VI
12	21	Female	WASSCE	Christianity	Enugu	Igbo	PD
13	19	Male	FSLC	Christianity	Taraba	Hausa	PD
14	26	Female	FSLC	Islam	Anambra	Igbo	PD
15	31	Female	WASSCE	Islam	Taraba	Hausa	PD
16	24	Female	FSLC	Christianity	Enugu	Igbo	VI
17	35	Female	M.SW	Christianity	Enugu	Igbo	ND
18	29	Male	M.SW	Christianity	Enugu	Igbo	PD
19	48	Female	M.SW	Christianity	Enugu	Igbo	ND
20	42	Female	M.SW	Christianity	Anambra	Igbo	ND

S/N 1–16 were persons with disability, and S/N 17–20 were social workers; FSLC, First School Leaving Certificate (certificate issued after completing 6 years of primary education); WASSCE, West African Senior Secondary Certificate Examination (certificate issued after completing 6 years of secondary school); M. SW, Master of Social Work; B.Sc., Bachelor of Science degree; PD, Physical disability; VI, Visual impairment; ND, No disability.

### 3.1 Mistrust of the government

The concept of trust and mistrust emerged throughout the participant’s statements. As described by the participants that contribute to their mistrust, several factors include a high level of corruption in the government, historically, government lack of concern for citizen welfare, especially PWD, lack of social inclusion, and the non-implementation of Disability act in most Nigerian states. The participants did not trust the government because of their belief that government is corrupt, and the COVID-19 pandemic creates another avenue for the government to enrich itself. Others believed that the COVID-19 lockdown by the government was only a political game to attract international relief funds. Below are narratives from participants to illustrate this theme.

*“Our government is corrupt and would do anything to attract funds from world health organizations and other international agencies. I believe that the government orchestrates the lockdown in Nigeria and the increasing number of recorded cases of Coronavirus to attract foreign sympathy and supports.”* (Female, PWD-7, 20 years old).

PWD believed that historically, the government does not support her citizen’s welfare, specifically PWD. Hence, they believe that the palliative received by the international agencies will not reach its citizens.

*“To clarify that the government does not have our interest at heart, they locked down places and forgot that people like me and other persons with disabilities in institutions need to be provided for in such homes. Palliatives from the government have been more on paper without an objective implementation.”* (Female, PWD-6, 27 years old).

PWDs and social workers were skeptical concerning the proposed COVID-19 palliatives because Nigeria Disability Act 2018 has only been implemented in 6 out of the 36 states since its introduction by the Nigerian Federal government. This further increases their mistrust of the government.

*“The Nigerian federal government has enacted the Nigerian Disability Act; however, only about 6 out of 36 states in Nigeria have partially implemented it. The lack of implementation (even partially) of this Act continues to raise questions and doubts over the governments.”* (Female, Social worker-17, 35 years).

The mistrust of the government appears to contribute to the non-adherence of the COVID-19-pandemic strategies, especially lockdown, as it has more negative impact than positive on their health and wellbeing.

*“Mistrust for the government cuts across persons with a disability like myself. If we do not trust the government, how then should we believe them that COVID-19 is actually in Nigeria’? we need to have that trust and belief to be able to accept and obey the preventive measures by the government.”* (Female, PWD-9, 22 years old).

*“Nigerians generally and PWD specifically have many reasons for their mistrust of the Nigerian government, and this is one of the reasons I feel they have not taken the COVID-19 prevention warnings of the government seriously.”* (Male Social worker-18, 29 years old).

### 3.2 Conspiracy theories perpetuated by the media and religious and cultural beliefs

All PWDs believed that Nigerians are immune to the virus and berated the Nigerian government for the lockdown. More worrisome is the assumption among participants that COVID-19 is a sickness of the elites and bourgeoisie with abroad travel histories.

*“…have you seen any media report about a poor man with COVID-19? All reports of COVID-19 cases I have heard of are of the rich and influential persons in Abuja and Lagos who have the resources to travel worldwide.*” (Female, PWD, P14, 26 years old).

One of the social workers collaborated on this assumption held by the PWDs.

*“Up to a month after the outbreak of COVID-19 in China, almost none of my patients believed that the virus was real more especially as a lesser number of cases are reported in Nigeria. Even with the record of COVID-19 cases in Nigeria and associated deaths, most of my patients continue to believe that the virus is not real and at best believe it is a virus for the rich because of their penchant to travel to foreign countries for business, medical, leisure or* politics.” (Female, Social worker-19, 48 years old).

Some participants believed that COVID-19 is a weapon (5G network) developed by the devil to affect God’s children.

*“…Coronavirus cannot affect the children of God and those it affects are evil people who are facing punishment from the Supreme Being.”* (Female, PWD-7, 20 years old).

*“I believe COVID-19 is a punishment for those whose ways are sinful and not pleasing to Allah. I have also watched a video where a Christian pastor called Chris Oyakhilome said a similar thing and attributed the virus to technological development such as the 5G network.”* (Male, PWD-3, 25 years old).

We asked the social worker to describe their experience regarding their role in correcting the misconception held by the PWDs. Two social workers stated:

*“It is disconcerting that in this our dispensation of advanced science, people even some enlightened ones continue to think that the introduction of 5G network causes coronavirus. This is absurd, and to worsen the case, vaccine intake has been reportedly low among PWLD because they do not trust vaccines because of the misconceptions and beliefs.”* (Social worker-18, 29 years old).

*“Our first job is to correct the wrong assumptions people living with disability have. When I engaged this population, I noticed that they already have some image in their head about Coronavirus, and most of the information they hold are wrong perceptions…”* (Female, Social Worker-20, 42 years old).

### 3.3 Mistrust of government leading to poor compliance with COVID-19 preventive measures

Mixed reactions were observed on the compliance of COVID-19 safety measures. While respondents acknowledge the efforts of government and non-governmental agencies to enlighten the public on the safety measures to prevent the spread of COVID-19, they, however, were not comfortable with such rules. Most of the non-compliance stems from the non-belief that COVID-19 exists.

*“I do not believe that this virus exists, so why should I suffocate myself with a face mask in the name of compliance with government directives?”* (Male, PWD-3, 25 years old).

#### 3.3.1 Obeying some of the rules to please doctors and family members

Most participants stated that they obeyed some of the rules for COVID-19 prevention measures either to please their doctor or family members.

*“… After the ease of the first phase of the lockdown, I used a face mask not because I believed in the existence of the virus or its ability to prevent the spread of the virus. I only used it to satisfy the doctors who would not come close to treating patients or check on their wellbeing if they were not wearing masks. They usually stayed away from patients and asked us to put on our masks.”* (Female PWD-4, 24 years old).

Further, some participants stated that culturally in Nigeria, distancing oneself from another is not ideal. Most of them socialized with friends and families by seeking opportunities to attend social events (e.g., parties and church services).

*“How can we live or survive in isolation or without the company of others……… how possible is it for us to stay away from each other or fully maintain the two meters’ distance? Parties and church services are the easiest way for us to forget the hardship in this country… asking us to stay apart, even though we are yet to believe this COVID-19 exists is very difficult.”* (Male PWD-3, 25 years old).

#### 3.3.2 Hand washing and sanitizing is the routine practice in the homes

Amongst all preventive measures, participants were more likely to engage in handwashing and hand sanitation. However, this measure is seen as a routine practice in the rehabilitation home rather than COVID-19 prevention compliance.

*“… The management of this home is particular about compliance with the COVID-19 preventive measures, and they provided all the necessary facilities to ensure compliance, for instance, the solar energy-powered hand washing machine and sanitizer dispenser. However, if I must be frank with you, most people here wash their hands and use sanitizer because we are used to it and not necessarily because of the virus prevention. After all, before Coronavirus came, we do wash our hands when we feel like they are dirty.”* (Female PWD-8, 18 years old).

#### 3.3.3 Increased doubt over COVID-19 vaccine uptake

Participants remain unsure about the uptake of the COVID-19 vaccine for fears such as its efficacy and possible complications. Participants reported that lack of trust in the government is the major issue that could prevent them from taking the vaccine. They do not know what is in the vaccine and what the government is planning.

*“The Government has not shown serious care about us. Why then should we believe their supposed care now? I have an underlining condition already, and I should be even more careful with what I take into my system-I mean the COVID-19 vaccine. Because I do not trust the intention of the Government regarding COVID-19 in Nigeria, I seriously doubt I will take the vaccine when available.”*(Female, PWD-8 18 years old)

Other factors that may hinder PWDs from taking the vaccine may include proximity to the vaccine centers.

*“We expect the vaccine to be brought to us in our residential facility, but we doubt if that will happen in Nigeria where our welfare is not fully prioritized, and this will add to the reasons I may personally not take the COVID-19 vaccine…”* (Male, PWD-13, 19 years).

The participants believed that some of them had not been adequately educated on the advantages and disadvantages of taking the vaccine. They believed that there could be complications, especially for people with other health conditions or low immunity.

*“I cannot take the vaccine because I have no symptom of COVID-19, and there is a rumor that some older people who have taken it are having some complications. I cannot attempt to add salt to my injury. I have health issues I am battling with already, and I cannot complicate things for myself until the vaccine is proven tasted and trusted.”* (Male, PWD-2, 27 years).

### 3.4 Pandemic restrictions slowed healing and subsequent limited access to healthcare and vaccination

#### 3.4.1 Delayed physical healing contributes to accessibility barrier in healthcare and vaccination

PWDs in Nigeria faced significant challenges during the COVID-19 pandemic, particularly due to lockdown restrictions. These measures not only exacerbated hunger and socioeconomic hardship but also delayed access to healthcare services, including vaccination. The closure of businesses and charitable organizations, a primary source of support for many PWD, made basic survival difficult. As a result, financial constraints further delayed their access to essential medical care and rehabilitation, impeding physical recovery.

For example, one participant shared how the lockdown affected their access to surgery-related follow-up care due to a lack of funds:

*“My orthopedic amputation surgery was successful, and I was healing well. But my parents couldn’t pay the medical bills because their business was shut down during the lockdown. Not only healthcare, even feeding became a struggle; and with the uncertainty of the pandemic, those who could help were also hesitant.”* (Male PWD, 23 years old).

This lack of financial resources and mobility restricted access to healthcare services and, subsequently, COVID-19 vaccines. Many PWDs were unable to travel to healthcare facilities due to reduced income and movement restrictions, further limiting vaccination uptake.

For many PWDs, physical impairments already posed significant barriers to accessing healthcare, and the pandemic further complicated these challenges. The inability of medical personnel to provide hands-on care due to social distancing requirements left many PWDs without the necessary support for their recovery. One participant described the struggle:

*“Even before the pandemic, moving around for medical care was difficult due to my condition. Now, with the restrictions, medical staffs maintain more distance, it feels like getting the help I need has become almost impossible.”* (Female PWD, 18 years old).

#### 3.4.2 Reduced spiritual support impacts mental well-being and subsequent health-seeking

In addition to physical challenges, many PWDs experienced a delay in their perceived spiritual and emotional healing due to the closure of religious institutions. For individuals who rely on faith for healing, the lack of access to religious gatherings created a sense of spiritual isolation, impacting their overall well-being and health management. This added emotional burden, combined with physical pain, limited their ability to actively seek out healthcare and vaccination services.

One participant highlighted the role of religious support in their healing process:

*“…… not being able to visit or have my pastor visit me for prayers could be part of the reasons I continue to feel some physical pains. I continue to be worried; I don't feel like doing anything else for my health…”* (Female PWD, 21 years old).

Overall, the pandemic’s restrictions created a cycle of delayed recovery, worsened living conditions, and limited access to healthcare including vaccines, as many PWDs found it difficult to overcome both physical and logistical barriers during this period.

### 3.5 Roles of social workers in supporting PWDs and increasing COVID-19 vaccination uptake during the pandemic

The COVID-19 pandemic not only exposed existing vulnerabilities among persons with disabilities (PWDs) but also provided an opportunity for social workers, especially those in medical and disability services, to step up and expand their roles. Their involvement, both voluntary and professional, addressed critical gaps in healthcare access, psychosocial health, and overall well-being, which became more acute during the pandemic. Merging their experiences from the in-depth interviews, revealed the multi-dimensional impact of social worker interventions, and provided a deeper insight into the value they bring and the systemic changes needed to better support PWDs.

Analyzing the role of social workers for people with disabilities (PWDs) during the pandemic has revealed four key dimensions of their work, as presented in Table 3. These dimensions are discussed below.

**Table 3:** Roles of social workers in supporting persons with disabilities during the COVID-19 pandemic: dimensions and key tasks.

Dimensions	Tasks
Adaptation and flexibility	Expanded on new roles, volunteered on weekends
Psychosocial support	Provided counseling, offered psychotherapy, worked toward reducing vaccine-related fear
Advocacy for systemic change	Lobbied for funding and better healthcare including vaccination access, called for inclusive policies
Building resilience and trust	Educated on vaccines and virus spread, promoted preventive practices

#### 3.5.1 Adaptation and flexibility of social workers

One key dimension that emerged during the pandemic was the adaptability of social workers to the unprecedented challenges PWDs faced. As traditional support systems were disrupted due to lockdowns and physical distancing measures, social workers had to rapidly adjust, adopting new roles and responsibilities to ensure that PWDs received the necessary support.

*“COVID-19 pandemic and lockdown gave some of us the ample opportunity to sell ourselves and show our expertise as social workers…. Colleagues who embraced this new opportunity during the pandemic are mostly those who are medical or disability specialists. Some of us even went as far as volunteering for other agencies and rehabilitation homes on weekends. I must tell you that the relatively few people we worked with have come to appreciate what we do as social workers.”* (Female Social worker-17, 35 years old).

Specific job roles identified by social workers that they engaged in to assist people living with a disability included, but were not limited to Community Surveillance Officers, In-house Educators, Community Referral Officers, and Engagement Officers. As part of these roles, they contributed in monitoring PWDs for COVID-19 symptoms, educating PWDs about vaccine efficacy and debunking myths, facilitating referrals to COVID-19 vaccination facilities, and ensuring equitable distribution of healthcare resources including vaccines. Notably, the social workers created these roles in the rehabilitation homes to demonstrate the social worker’s role during the COVID-19 pandemic.

The volunteering efforts of social workers went beyond the call of duty, highlighting the profession’s inherent flexibility and dedication to human welfare. The creation of these new roles exemplified how social workers adapted to meet the needs of their communities in real-time, showcasing a holistic approach to disability support.

*“All of us have our day jobs; we just volunteer most evenings and weekends on these roles”* (Male Social worker-18, 29 years old).

#### 3.5.2 Psychosocial support for PWDs

Another critical dimension is the psychosocial impact of the pandemic on PWDs. Many PWDs experienced heightened psychosocial health challenges due to isolation, uncertainty, and economic hardship. Social workers stepped in to provide essential counseling and psychotherapy services, addressing issues such as depression, anxiety, and trauma induced by the pandemic.

*“Generally, social workers are focused on human wellbeing, which has different biological, psychological, and social wellbeing dimensions. Therefore, our job role is quite vast, to sum up. For the sake of specificity, in some of the job roles I did to support PWD during the lockdown with counseling and psychotherapy.”* (Female Social worker, 20 years old).

This dimension reveals the role of social workers extended beyond direct physical care to encompass psychosocial support, demonstrating social workers engagement in addressing the full spectrum of well-being, including psychological resilience toward fear of vaccine.

#### 3.5.3 Advocacy for systemic change

Social workers also informed about their advocacy roles, particularly in pushing for systemic changes that benefit PWDs. They lobbied to attract funds and palliative care to various institutions they worked with during the pandemic. Additionally, they used their platforms to call for disability-inclusive policies and programs, both at the governmental and institutional levels. Their advocacy efforts included call for better vaccination and healthcare access, financial assistance, and targeted vaccinations and interventions for PWDs during the pandemic.

*“Our advocacy works included but not limited to lobbying for funds and palliatives, ensuring equitable distribution of vaccines and other resources across institutions and among residents where available. I also advocate for the wellbeing of persons with disabilities in such time through the call for disability-inclusive policies and programs by the government and sensitization programs in different disability institutions”* (Female Social worker-20, 42 years old).

#### 3.5.4 Building community resilience and trust

In addition to providing direct services, social workers played a crucial role in building community resilience and trust among PWDs and their families. By serving as reliable sources of information and education, social workers were engaged in efforts to alleviate fears about the vaccine and the virus itself, and promoting preventive behaviors.

*“We created this role to help the PWD during this pandemic and educate them on COVID-19 vaccine. We took session in disability institutions on how Coronavirus spread and how to apply preventive measures in line with the World Health Organization.”* (Male Social worker, 29 years old).

This dimension reveals the key role of social workers in building community trust through their long-standing relationships with PWDs.

#### 3.5.5 Long-term implications and sustaining social worker engagement

Lastly, the pandemic’s impact on the social work profession itself is an important dimension to consider. The expanded roles and increased visibility expressed by the social workers during the crisis have demonstrated the critical importance of their work in times of emergency. However, their views also highlighted the need for better institutional support and recognition of social workers, who often operate in underfunded and understaffed environments.

*“We hope that the home will see the need of social workers and sustain the roles they play even after the COVID-pandemic”* (Male Social Worker, 29 years old).

*“Social workers are essential, especially in disability care, and our profession needs more support and resources.*” (Female Social Worker, 42 years old).

This dimension underscores the long-term implications for the profession include the need for formal recognition of expanded roles and improved funding for social work services. A concerted opinion of the social workers was that the pandemic has shown their value in supporting vulnerable populations, and this should be reflected in policies and funding structures moving forward.

## 4 Discussion

This study explored how PWDs’ perceptions of the COVID-19 pandemic informed vaccine uptake in Nigerian care institutions and the social workers’ perception of their role in increasing vaccine uptake among PWDs in rehabilitation homes. Our study noted that participants’ mistrust of government, conspiracy theories perpetuated by the media, and participants’ culture and religious beliefs influence their poor compliance with COVID-19 prevention strategies, leading to increased doubt over COVID-19 vaccine uptake.

PWDs’ perceptions were guided by the public perception of conspiracy theories that influence their beliefs on the COVID-19 pandemic and vaccine uptake. The belief in conspiracy theories (e.g., the 5G network conspiracy theory) is associated with negative public health behavior, such as the unwillingness to social distance and vaccinate against the virus (34). Furthermore, some religious doctrines in Nigeria emphasize faith healing over medical intervention, leading to skepticism about vaccines. Additionally, there are infrequent but deep-rooted cultural norms that value traditional medicine and distrust Western medical practices, which contribute to vaccine hesitancy. The exposure of PWDs to various cultural and religious beliefs regarding their physical conditions may further compound their mistrust in the COVID-19 vaccine, as these beliefs often intersect with broader societal skepticism and conspiracy theories perpetuated by the media. Although not among PWD, Romer and Jameison (35) conducted a national survey and reported that belief in three COVID-19 Related conspiracy theories was inversely related to the perceived threat of a pandemic, taking preventive actions (e.g., wearing a face mask), perceived safety of vaccination and intention to be vaccinated against COVID-19. Global studies have also highlighted comparable challenges regarding vaccine uptake among PWDs. Although there has been limited research among this population, studies in diverse settings have identified low vaccine uptake and high vaccine hesitancy among PWDs compared to the general population (36, 37), with lack of vaccine education and misconceptions playing a vital part (37–39). Additionally, global evidence has underscored the influence of socioeconomic factors and accessibility barriers on vaccine hesitancy among PWDs, emphasizing the need to strengthen the roles of caregivers and social workers (40, 41). These findings suggest that while specific cultural and social practices influences may vary locally, the broader trend of these factors contributing to vaccine hesitancy among PWDs is pervasive globally, necessitating context-specific strategies to enhance vaccine acceptance. Since belief in conspiracy theories is a strong predictor for vaccine uptake, we argued that re-educating the PWDs with global evidence on the causes and the importance of the COVID-19 vaccine should be prioritized in the Nigerian context. This education can be achieved with social workers who have created volunteer positions (e.g., community surveillance officer, in-house educator) in the rehabilitation homes.

PWDs perceived that the COVID-19 lockdown impacted their general wellbeing as their religious and socioeconomic life suffered. PWDs depend on social and religious gatherings to socialize outside the rehabilitation home (42, 43). These social and religious gatherings were shut down because of the nationwide lockdown. Most PWDs depend on other people such as caregivers, family, friends, philanthropists for their livelihood and daily survival. This finding agrees with McKibbin and Fernando’s (44) and Guerrieri et al.’s (45) findings that COVID-19 lockdown has affected the developed economies and has brought untold economic hardship among people, especially those who live on daily wages. This economic hardship is worst for PWDs in developing countries, as strategies or processes to provide palliatives and government subsidies or emergency funds are either lacking or poorly managed (46). PWDs depend on international, national, and individual donations in Nigeria and some developing countries (47). However, these donations, especially individual’s donations, are affected by the Lockdown, thereby increasing the hardship experienced by PWDs in the rehabilitation homes.

Although PWDs often do not comply with all the COVID-19 preventive measures, they stated that hand washing and the use of sanitizer is not new in their rehabilitation homes, as it has been standard practice in their home even before the arrival of COVID-19. Generally, PWDs’ compliance to COVID-19 preventive measures is low, as it can be linked to their mistrust in government and misconception about COVID-19, including that COVID-19, does not exist. This low compliance to COVID-19 safety measures is not peculiar to PWD (48, 49), but it is concerning as they [PWD] are at higher risk of contracting COVID-19. Interestingly, social workers in our study reported that one of their roles since the pandemic is to educate PWD on the importance of obeying COVID-19 prevention strategies, as evidence has shown that targeted education is promising to increase compliance (50, 51). Our respondents were unsure about taking the COVID-19 vaccine, given reasons for mistrust of the government and the proximity of vaccine uptake centers, and some fears regarding the vaccine’s efficacy.

Our study’s secondary aim was to explore the social workers’ perception of their role in increasing vaccine uptake among PWDs in rehabilitation homes in Nigerian. Social workers have been continually active since the COVID-19 pandemic, including the lockdown period in Nigeria. Social workers specializing in medical and disability fields have seized the opportunity to establish their professional roles with PWD residents in health and social care institutions. Prior to COVID-19, Nigerian social workers’ roles were overtly ignored or not acknowledged by the government or the citizens (52). Social workers who participated in our studies created innovative positions in the rehabilitation homes to educate the PWDs on the COVID-19 pandemic, its strategies, and the importance of vaccine uptake. Our study’s social workers’ roles were volunteered grassroots roles, e.g., community sensitization officer that works directly with PWD. These roles lessen the negative impact of lockdown on PWD, as most social workers’ role is for psychological readjustment and strengthening PWD’s ability to cope with the ‘new normal’ (53). However, our findings did not provide evidence on the social workers’ role in education to increase the COVID-19 vaccine. There could be several reasons for this. The study interview was conducted when the lockdown was in place, social workers emphasized promoting mental health among PWDs, and the COVID-19 vaccine was at the development stage. Regardless, these active roles describe by our social worker participants are congruent with previous findings that social workers also provide counseling and referral services to clients and advocate for providing essential services for their wellbeing (48). The findings of other studies are not different from this present study on the place of social workers during the lockdown. For example, the International Federation of Social Workers [IFSW] (25) confirmed that social workers render essential services necessary during social upheavals like this present pandemic and lockdown. Brindle (54) further found that social workers are currently creating safety measures for vulnerable communities such as PWD to manage COVID-19 and avoid its further spread.

### 4.1 Study limitations and strengths

Although our study has provided insights into PWDs’ perception of COVID-19 and vaccine uptake, we must acknowledge some limitations. We did not perform participants’ member checking, as this approach would have increased the strength of our study findings. Since the current study was conducted among PWDs in institutions using a qualitative interview format, the sample size involved may be considered small; hence we caution the generalization of our findings to the general population of PWDs. Future research can enhance the understanding and generalizability of findings by employing a mixed methods approach that combines representative quantitative surveys with alternative qualitative components like focus group discussions (FGDs). In a mixed-methods approach, quantitative surveys could capture broad trends and demographics influencing vaccine uptake, while FGDs could delve deeper into nuanced perspectives and community-specific concerns regarding COVID-19 vaccine among PWDs. Furthermore, utilizing a representative sample of PWDs from both the community and institutional settings would provide a more comprehensive understanding of vaccine perceptions. Moreover, future studies can consider exploring the differences in the perception of the COVID-19 vaccine across different PWDs, for instance, people living with physical disabilities and people with intellectual disabilities.

As for strengths, our study employed peer member checking, we maintained reflexivity by identifying our “Subjective I” to enhance rigor, and we increased this study’s credibility by using double coders at each stage of our analysis.

## 5 Conclusion

Participants’ mistrust of government, conspiracy theories perpetuated by the media, and participants’ culture and religious beliefs influence their poor compliance with COVID-19 prevention strategies, leading to increased doubt over COVID-19 vaccine uptake. PWDs residing in three rehabilitation homes in Nigeria believe that COVID-19 does not exist mainly due to mistrust in government; thus, they would not comply with the evidence-based preventive measures. Interestingly, hand washing, and hand sanitizer were practiced because it is the rehabilitation home culture, but not as the other COVID-19 measure, including social distancing. Amid the lack of institutionalization of the social work profession in Nigeria, social workers have continued to show their relevance to the Nigerian community during COVID-19, especially among PWD. Social workers created grassroots volunteer positions that allow them to work directly with PWD in their rehabilitation homes.

Based on our study findings, we recommend that government and NGOs employ targeted communication strategies to address the cultural and religious beliefs contributing to vaccine hesitancy among PWDs, involving trusted community leaders. Educational programs providing clear, factual information about COVID-19 and vaccines should counteract misinformation and conspiracy theories, using formats such as workshops, leaflets, and digital content. To enhance the role of social workers, the government should devise structured pathways for their support to PWDs in both institutional and community settings during pandemics. Future implementation research should explore the best strategies and practices for their integration and impact on health outcomes for PWDs. Future research and policy advocacy should focus on generating robust evidence on the role of social workers and establishing educational and paid support systems for them, promoting their institutionalization in Nigeria and similar contexts during pandemic-like crises.

## Data Availability

Transcripts of the interviews will be available upon reasonable request from the corresponding author.
