# Supplementary material for: Connecting the experiences of persons with disabilities and social workers in Nigerian care institutions regarding COVID-19 vaccine uptake: a qualitative descriptive-interpretive design
Source: Front Public Health. 2024 Oct 9;12:1466313. doi: 10.3389/fpubh.2024.1466313 (PMC11496303; doi:10.3389/fpubh.2024.1466313)
Supplement: Supplementary file 1 [file Table_1.docx]

# Supplementary Material

**Supplementary file: Interview guide for PWD and social workers.**

| Interview guide for PWD to describe their perception of COVID-19 and their beliefs on the COVID-19 vaccine | Interview guide for social workers the social workers' perception of their role to increasing vaccine uptake among PWDs in rehabilitation homes. |
| --- | --- |
| 1. What do you understand by COVID-19? 2. What are your perceptions on COVID-19 in Nigeria?  - Probe for accuracy - Probe for depth and source  1. What are your beliefs on the COVID-19 preventive measures?  - Probe for non-pharmaceutical measures, e.g., face mask, washing of hands, etc - Probe for pharmaceutical measures, e.g., COVID-19 vaccine intake. - Why is it? - Why not?  1. Did the first lockdown in Nigeria affect your life?  - Probe for health impact - Probe for socioeconomic impact | 1. What are your roles towards persons living with disabilities?  - Probe for job function  1. For how long have you been working with persons living with disabilities? 2. What do you think are the perceptions of PWD on COVID-19 preventive measures?  - Probe for perceptions on lockdown - Probe for perceptions on COVID-19 vaccine uptake  1. How has covid19 affected your job function with persons living with disabilities? 2. Do you think the first lockdown in Nigeria impacted the wellbeing of PWD?  - Can you describe the positive or negative impact, if any? - Why not?  1. What are your perceptions of the role of social workers in helping PWD in the first lockdown in Nigeria? 2. What should social workers have done to help persons living with disabilities to cushion the effect of covid19 on them?  - Probe for policies advocacy - Probe for palliative distribution |
